# Tracking Current Evidence and Future Perspectives of Children’s Motor Development

**DOI:** 10.3390/children13040492

**Published:** 2026-03-31

**Authors:** Domenico Monacis, Dario Colella

**Affiliations:** 1 Department of Education and Sports Sciences, Pegaso University, 80143 Naples, Italy; 2 Department of Biological and Environmental Sciences and Technologies, University of Salento, 73100 Lecce, Italy; dario.colella@unisalento.it

Motor development is a fundamental and multifaceted pillar of a child’s developmental journey, serving as a fundamental foundation for optimizing a child’s physical, cognitive, emotional, and psychosocial well-being [1]. In today’s social and healthcare landscape, characterized by a concerning prevalence of hypokinetic phenotypes and growing challenges related to the heterogeneous trajectories of neurodevelopment [2,3], the academic community is called upon to produce robust empirical evidence. This body of evidence is essential for guiding intervention strategies that promote Physical Literacy, active lifestyles, foster full inclusion, and maximize each individual’s developmental potential, moving beyond purely compensatory or therapeutically approaches.

This Special Issue, titled “*Recent Advances in Children’s Motor Development: From Birth to Adolescence*,” compiles nine high-profile scientific contributions, presenting a significant synergic and innovative epistemological framework. The primary added value of this Special Issue lies in the dense network of theoretical and methodological interconnections among the published studies, which, while investigating different populations and developmental stages, converge toward the overcoming of biomechanical reductionism in favor of a holistic, ecological, and embodied construct.

Within this context of epistemological renewal, physical experiences in the fields of physical education, extracurricular activities, and sports initiation are not merely an indispensable component of the educational curriculum, but a genuine ecosystem for interdisciplinary intervention [4]. However, driven by current socio-cultural transformations—primarily the pervasive integration of digital tools and devices in childhood—the ways in which children access knowledge and interact with their peers are changing rapidly. In this evolving landscape, the translation of developmental theory into educational practice often reveals methodological opacity, especially in managing the delicate transition from spontaneous movement to structured sports practice in the absence of solid pedagogical foundations [4]. Although the scientific literature of the past decade shows a growing focus on the prevention of hypokinesia, health promotion, and the optimization of motor learning processes, we are simultaneously witnessing a progressive fragmentation and decontextualization of best practices oriented toward motor development [5]. In a society that tends to compress, overstructure, or even suppress certain dimensions of motor development in favor of partial, formal, and adult-centered educational approaches, there is an urgent need to embrace pedagogical models that reflect the complex, fluid, and context-dependent nature of developmental change.

It is crucial to restore centrality to a motor experience that is intrinsically motivating, spontaneous, creative, and radically inclusive, specifically designed to enhance the deep interconnection between perception, action, cognition, and physical fitness [6]. Movement, in fact, represents a pervasive, foundational, and highly transformative phenomenon in a child’s ontogenesis. It acts as an irreplaceable medium for navigating different contexts (e.g., school, sports, and leisure time) and for translating theory into practice, long before motor actions take on the conventional, strictly utilitarian, and rational connotations typical of adulthood [4,5].

In perfect alignment with the aims of this Special Issue, it is crucial to reaffirm the educational value of movement-based experiences, with a particular emphasis on pedagogical and health-related models to lifespan motor development, catalyzing and nurturing those cognitive–motor, affective, and relational interconnections that constitute the very essence of harmonious and holistic development [7,8].

Below, we offer a critical and cross-cutting examination of the contributions, highlighting their innovative contribution to the advancement of research on motor development.

1. *The Developmental Ecosystem: Early Determinants and School Dynamics*

A central pillar of this Special Issue is the adoption of an ecological paradigm, which posits that the acquisition of motor skills is inextricably linked to environmental variables. The study by An and Libertus provides the foundation for this perspective, demonstrating, through an Integrated Ecological Model, how parental constructs (beliefs, attitudes, and expectations) significantly modulate the very earliest neuromotor trajectories of the newborn.

This fundamental focus on the developmental ecosystem finds its natural extension in the context of formal education through the study by Luptáková et al., which analyzes the effectiveness of co-teaching models (Team Teaching) in primary physical education classes. Both studies suggest significant pedagogical implications for driving educational interventions: the optimization of children’s motor development cannot be separated from the structuring of qualified learning environments and the training of caregivers (parents and teachers). To evaluate the effectiveness of such settings, an ecologically valid psychometric assessment tool was needed. In this regard, Iannaccone et al. provide the cross-cultural validation of the CAPL-2 questionnaire for the Italian children, allowing for the assessment of Physical Literacy by integrating the performance of motor tasks with affective–cognitive dimensions, such as motivation and perceived competence.

2. *Neurocognitive Synergies: Epistemological Continuity Between Developmental Disorders and High Performance*

The most innovative heuristic approach in this Special Issue emerges from a joint examination of Han et al.’s review on cluttering and Timea et al.’s controlled trial on adolescent athletes. Although situated at opposite ends of the spectrum of functional abilities, these studies share a primary neurophysiological focus: the intrinsic interdependence between executive functions and motor control.

Han and colleagues reconceptualize alterations in verbal fluency not as a mere mechanical deficit, but rather as a specific vulnerability in motor–cognitive integration that manifests under conditions of high executive load. Similarly, Timea et al. address the same cognitive–motor interface in the context of elite athletic performance: the implementation of the SmartACT hybrid protocol (integrating mindfulness, cognitive flexibility, and visual–motor reaction technologies) demonstrates that training in executive functions and the mitigation of psychological stress are direct predictors of optimized agility and reaction times. A significant paradigm shift emerges from these findings: the cognitive–motor interface constitutes the primary target for intervention, whether aimed at supporting neurotypical development, managing atypical conditions, or enhancing performance.

3. *Movement as an Immersive Medium for Inclusion and Systemic Adaptation*

A third thematic strand reinforces the view of movement as a vehicle for global adaptation, focusing on the quality of the motor experience rather than its mere quantification. The contributions by Lev-Ari et al. and by Lotan and Weiss, centered on individuals with Autism Spectrum Disorder (ASD), deconstruct isolated approaches in favor of immersive ecosystems. The practice of Capoeira (Lev-Ari et al.) and hydrotherapy protocols (Lotan and Weiss) emerge as complex socio-sensory environments capable of best promoting postural regulation, balance, and socio-communicative engagement, acting as powerful inclusive catalysts.

This conceptualization of movement as a promoter of systemic health finds full analytical support in the study by Monacis et al. Developing both mediation and moderation models in secondary schoolchildren (aged 11–14 years old), the authors corroborate the hypothesis that Physical Activity Levels (PALs) act as essential mediators between Body Mass Index (BMI) and physical fitness development. The key finding is that structured and consistent physical engagement has the capacity to mitigate the potential functional limitations associated with excess weight. Underlying these optimization processes is the need for proper stratification of neuromotor risk: in this regard, the contribution by Podstawski et al. on the use of the Vojta method reaffirms the importance of early identification of central coordination disorders, an indispensable prerequisite for the timely initiation of harmonious motor development pathways.

4. *Concluding Reflections and Future Research Perspectives*

The nine studies presented here confirm that the motor development process must be investigated as a complex dynamic system, resulting from the continuous interaction between the organism’s neurobiological constraints, the affordances of the physical and relational environment, and the task constraints of specific human activities.

In order to improve our understanding of recent advancements in motor development, future research should focus on the following strategic areas:

1. Longitudinal and multicenter designs: Tracking motor development trajectories over the long term and investigating how early determinants and parenting styles predict adherence to physical activity and motor competence in adulthood;
2. Technology assessment tools: advances in wearable technologies and artificial intelligence must be leveraged to collect kinematic and behavioral data in real-world settings, overcoming the inherent limitations of laboratory evaluations and self-report measures;
3. Transdisciplinary synergy: promoting a research framework that structurally integrates cognitive neuroscience, movement sciences, and educational disciplines, in order to validate multi-component interventions that support the well-documented link between motor skills, executive functions, and emotional domains;
4. Translation into health policy and education paradigms: the robust evidence emerging from this Special Issue must be translated into stringent policy guidelines, incentivizing structural presence of movement specialists starting in elementary school and promoting the integration of inclusive motor experiences (such as adapted martial arts or aquatic activities) into the Essential Levels of Care (LEAs) and public health promotion programs.

In closing, as Guest Editors, we want to thank the authors for sharing their research.

We also want to thank the peer reviewers for checking this research; their feedback was very helpful. We also thank the Editorial Board of *Children* for always helping us. We think this collection of research will be very useful for scholars, teachers and the international academic community.
